# ASO Author Reflections: From Recommendation to Reality: Expert Multidisciplinary Collaboration in Intrahepatic Cholangiocarcinoma Care

**DOI:** 10.1245/s10434-026-19687-x

**Published:** 2026-04-24

**Authors:** Thomas C. Zwaan, Stefan Buettner, Bas Groot Koerkamp, Pim B. Olthof

**Affiliations:** 1 Department of Surgery, Erasmus MC Cancer Institute, Rotterdam, The Netherlands; 2 Department of Hepatobiliary, Transplant and Endocrine Surgery, Universiteitsziekenhuis, Antwerpen, Antwerp, Belgium

## Past

Intrahepatic cholangiocarcinoma (iCCA) is a rare and aggressive malignancy, often diagnosed at an advanced stage and associated with poor survival. Although surgical resection offers the only chance for long-term survival, only a minority of patients are eligible.^1^ Current guidelines recommend multidisciplinary evaluation, preferably involving expert centers, to optimize staging and treatment selection.^2,3^ Despite an existing centralized approach of care for patients with iCCA in the Netherlands, variation in referral patterns and multidisciplinary team (MDT) participation persists. Real-world evidence demonstrating the impact of expert MDT involvement on outcomes in iCCA has been limited.

## Present

In this nationwide study of 1622 patients with iCCA in the Netherlands, discussion with an expert center was independently associated with improved overall survival. Patients discussed in an expert MDT were more likely to receive tumor-directed therapy and undergo resection, while those diagnosed in expert centers had higher resection rates.^4^ These findings suggest that expert multidisciplinary evaluation expands treatment opportunities and improves outcomes, consistent with prior evidence demonstrating the benefit of multidisciplinary care in oncology.^5^ Survival differences were most pronounced early after diagnosis, indicating that patient selection and unmeasured factors may partly contribute. In addition, substantial regional variation in MDT participation and management was observed, highlighting inconsistent implementation of expert collaboration.

## Future

These results emphasize the need to move from guideline recommendations to consistent implementation. All patients with iCCA should be considered for discussion in an expert MDT, regardless of disease stage or hospital of presentation. Efforts should focus on optimizing referral pathways, reducing regional variation, and strengthening collaboration between expert and non-expert centers. Future research should address barriers to referral, while expanding access to clinical trials and specialized treatments through expert centers, as this access remains essential. Timely referral to an expert MDT should be a standard step in iCCA care to improve outcomes.
